# Microwave-Assisted Extraction of Pectin from “Saba” Banana Peel Waste: Optimization, Characterization, and Rheology Study

**DOI:** 10.1155/2020/8879425

**Published:** 2020-11-24

**Authors:** Joel P. Rivadeneira, Tao Wu, Quincy Ybanez, Adeliza A. Dorado, Veronica P. Migo, Fidel Rey P. Nayve, Katherine Ann T. Castillo-Israel

**Affiliations:** ^1^Department of Food Science, University of Tennessee, Knoxville 37998, USA; ^2^Institute of Food Science and Technology, University of the Philippines Los Banos, Laguna 4031, Philippines; ^3^Agricultural Systems Institute, University of the Philippines Los Banos, Laguna 4031, Philippines; ^4^Department of Chemical Engineering, University of the Philippines Los Banos, Laguna 4031, Philippines; ^5^National Institute of Molecular Biology and Biotechnology, University of the Philippines Los Banos, Laguna 4031, Philippines

## Abstract

One way to valorize “Saba” banana peel waste is to extract high-value compounds, such as pectin, and use it for food applications. In this study, the parameters for the microwave-assisted extraction of pectin were screened and optimized using Response Surface Methodology. The pectin was purified and then subjected to characterization. Results showed that the optimum extraction conditions were 195°C, 8% solid-liquid ratio, and pH 3 hydrochloric acid (HCl), with predicted and actual yields of 12.8% and 14.2%, respectively. The subsequent purification method increased the purity of pectin by 300%. The pectin was found to be low-methoxy in nature and had an average particle size of 300 nm. The pectin application in whey protein isolate resulted in a shear-thinning fluid, with an improved viscosity compared to a control. When applied to a commercial orange juice, the *in vitro* digestion study showed that the fluid's viscosity was higher before and during the gastric and intestinal digestion at the low physiological shear rate.

## 1. Introduction

Banana is a locally-grown fruit in the Philippines that is available in the market year-round. It is a very abundant agricultural produce such that in 2019, the Philippines ranked second in terms of world export, next to Ecuador [1]. One of its most popular varieties is the “Saba” (Musa BBB “Saba” (*Musa acuminata x Musa balbisiana*)) cultivar, whose stem can attain a height of four meters. Simultaneously, its bunches can have 8 to 16 hands having 12 to 20 fingers per hand. The fruits are short, stubby, and highly angular with thick green peels when unripe and yellow peel when ripe. The pulp is creamy white, fine-textured with well-developed cores and occasional seeds. The “Saba” cultivar is usually consumed fresh in its ripened state and processed to provide variety to the food product.

According to the most recent Philippine survey, 25% of the country's net banana production, or around 2 million metric tons for 2019, undergo food processing [2]. The preliminary studies showed that after the processing, the waste produced is around two-thirds of the fresh material in terms of weight. Hence, the potential waste generation from banana processing is more than a million metric tons. Considering the volume of waste generated from the processing, which mostly ends up in landfills, the utilization of banana peels to create high-value products, such as pectin, is a good direction for research.

Pectin is used as a gelling agent, thickener, and stabilizer for food products like jams and jellies. Its application is also extended to beverages such as fruit juices and soft drinks. A significant advantage of adding pectin in food is that it reduces cooking time, improves texture and color, and increases the shelf-life. Aside from its function as a food ingredient, there were also claims of nutritional benefits from pectin. It is a soluble fiber that can draw water from the digestive system, forming a gel that helps slow down digestion.

Microwave-assisted extraction, one of the advanced ways of extracting pectin, has shown potential in saving time and energy and solvent consumption. The extraction of solutes from plant material using microwave involves penetration of solvent into the solid material, hydrolysis, diffusion to the surface, and external mass transfer to the solution [3]. In the hydrolysis phase, specifically, the energy absorbed by the plant tissue causes volumetric heating due to the dipole rotation of polar molecules, which causes molecular collision leading to heat generation inside the plant tissue [4]. Rapid heat generation due to microwave radiation also inactivates pectin esterase, an enzyme that interacts and reduces the solubility of pectic substances [5].

Studies involving microwave-assisted extraction of pectin have been performed for some substrates, including passion fruit [6], sour orange peel [7], and jackfruit rind [8]. The microwave power used to generate the high temperature (as a result of dipole rotation) needed for extraction ranged from 450-900 Watts. Meanwhile, the optimum extraction times for these substrates range from 2-20 min, shorter than conventional extraction using acidified water. Moreover, shorter exposure times were used for high microwave power (high temperature). Too long exposure to high-temperature results typically in the depolymerization/degradation of the hydrolyzed pectin, yielding polymers with a low degree of polymerization that does not precipitate in alcohol [9].

Given the abundance of banana peel waste and the understanding of its viability as a pectin source, it is an objective of this study to valorize the waste by extracting a high-value food ingredient from the banana peel. More importantly, the microwave-assisted extraction parameters were screened and optimized to maximize the yield of pectin—also, the properties of the pectin from banana peel waste. Finally, an actual application to a food product was performed to assess the pectin's performance in improving some food qualities.

The objective of this study is to evaluate and optimize the parameters (temperature, solid-liquid ratio (SLR), and pH) of the microwave-assisted extraction of pectin from “Saba” banana peel waste, characterize the pectin, and evaluate its thickening effect on beverages.

## 2. Materials and Methods

### 2.1. Materials

Fresh peels from mature unripe “Saba” banana were obtained from a banana chip processing plant in Santa Cruz, Marinduque, Philippines. It was sliced into small pieces (approximately 4 cm^2^) and then dried at 50°C until constant weight. The dried banana peel was powdered using a countertop blender (Oster®, Classic Series Accurate Blend™, Boca Raton, Florida) and stored in a desiccator at room temperature (25°C). Common chemicals and reagents, unless otherwise stated, were analytical reagent grade purchased from Fisher Scientific, USA.

### 2.2. Characterization

The moisture content, total ash, crude fat, and crude fiber were determined following the protocols described in the Official Methods of Analysis by The Association of Official Analytical Chemists [10].

### 2.3. Microwave-Assisted Extraction of “Saba” Banana Peel Waste

The extraction of pectin was done using a microwave digester (Ethos UP High-Performance Microwave Digestion System, Milestone Srl, Italy) with adjustable temperature and irradiation time. Depending on the SLR specified in the experimental design, powdered “Saba” banana peel waste was added to hydrochloric acid of different pH. The mixture was placed in the middle of the microwave digester's rotating disc and then exposed to varying temperatures within a specified length of time. Microwave power, the reserved energy, was set at 1,000 Watts. After the extraction process, the mixture was cooled to room temperature (25°C) and then filtered using a Grade 1 qualitative filter paper (11 *μ*m). The filtrate was separated, and an equal volume of 95% (*v*/*v*) ethanol was slowly added with continuous mixing. The mixture was incubated at 4°C for 2 h. The resulting microwave-extracted pectin (MEP) was recovered by filtration. It was washed twice with the same volume of 95% (*w*/*w*) ethanol and then dried at 40°C until constant weight. The percent yield was calculated using the following equation.

(1) $$\begin{array}{c} \%\text{Yield},\text{MEP}=\frac{\text{mass of crude pectin},\text{g}}{\text{mass of dried peel},\text{g}}\times 100. \end{array}$$

#### 2.3.1. Factorial Experiment for the Screening of Significant Extraction Parameters

Screening using a full-factorial experiment was performed to determine which of the microwave-assisted extraction parameters have significant main effects on the pectin yield. Four parameters: temperature (80-140°C), time (30-90 s), SLR (5-20% (*w*/*v*)), and pH (1-3) were evaluated.

#### 2.3.2. Optimization of the Microwave-Assisted Extraction of Pectin

A Central Composite Design was used to generate the combination of parameters for the microwave-assisted extraction of pectin from “Saba” banana peel waste. Three parameters: temperature (80-200°C), SLR (5-15% (*w*/*v*)), and pH (1-3) were evaluated. Meanwhile, the irradiation time for the extraction was standardized at 60 s.

### 2.4. Statistical Analysis

Design Expert Statistical Software 10 by Stat Ease, Inc. was used to run statistical tests for both the factorial and optimization experiments. For the earlier, the significant main effects of the microwave-assisted extraction parameters were chosen from the half-normal probability plot of the effects. At the same time, the corresponding magnitudes were obtained from the resulting Pareto chart. The significant parameters were validated with the consequent analysis of variance (ANOVA). For the latter, the software analyzed the responses using multiple regressions, and the resulting significance coefficients were evaluated using *F*-Test. A model was developed, and ANOVA determined its acceptability. Upon generating a valid model, the optimum combinations of extraction parameters were determined by numerical optimization.

#### 2.4.1. Validation of Optimum Microwave-Assisted Extraction Condition

The predicted optimum condition was validated by performing microwave-assisted extraction of pectin from “Saba” banana peel waste in triplicate and comparing the yield with the predicted value.

### 2.5. Purification of Microwave-Extracted Pectin (MEP)

Dried MEP (5% *w*/*v*) was dispersed in deionized water. The mixture was homogenized with a dispenser (Kinematica, Polytron 10-35 GT, Luzern, Switzerland) at 5,000 rpm for 5 min, and then stirred for 4 h. It was then centrifuged (Thermo Fisher Scientific, Sorvall Lynx 6000, Osterode, Germany) at 10,000 × *g* for 15 min. The supernatant was filtered with a Miracloth to remove any remaining residue. The filtrate volume was measured, and the purified microwave-extracted pectin (PMP) was precipitated by the addition of an equal volume of 95% ethanol. The mixture was centrifuged, and the PMP was collected by filtration using Miracloth. It was subjected to freeze-drying (Labconco, FreeZone™, Kansas City, Missouri), and the yield was calculated using the following equation.

(2) $$\begin{array}{c} \%\text{Yield},\text{PMP}=\frac{\text{mass PMP},\text{g}}{\text{ mass MEP},\text{g}}\times \%\text{Yield},\text{MEP}. \end{array}$$

### 2.6. Characterization of Pectin

#### 2.6.1. Methoxyl Content and Equivalent Weight

The equivalent weight and methoxyl content were measured using the method described by Rangana [11].

#### 2.6.2. Total Protein

The pectin's total protein was measured using the protocol described by Walker [12]. Solution A was prepared by dissolving 1 g sodium bicinchoninate (BCA), 2 g sodium carbonate, 0.16 g sodium tartrate, 0.4 g sodium hydroxide, and 0.95 g sodium bicarbonate in 80 mL deionized water. The solution was adjusted to pH 11.25 using 10 M sodium hydroxide and then diluted to 100 mL using deionized water. Solution B was prepared by dissolving 0.4 g copper sulfate pentahydrate in 8 mL water. The solution was then diluted to 10 mL. Solution C was made by combining solutions A and B at a 50 : 1 (*v*/*v*) ratio.

Two milliliters of solution C was added to a 0.1 mL sample, mixed using a vortex, then incubated at 60°C for 15 min. It was then cooled in an ice bath to reach room temperature (25°C). The absorbance was read against a blank (without sample) at 562 nm. For the standard, 0- 60 *μ*g of bovine serum albumin (BSA) was used. The concentration of the protein, in terms of BSA, was derived from the standard curve.

#### 2.6.3. Total Pectic Content

The total pectic content was measured following the method described by Blumenkrantz and Asboe-Hansen [13]. Seven and five-tenths milligrams of the sample was placed in a test tube containing a magnetic stir bar. The sample was placed in an ice bath, added with 5 mL cold sulfuric acid, and then stirred to allow the material's complete dissolution. A total of 1.25 mL of deionized water was slowly added, followed by continuous stirring for 5 min. Another 1.25 mL of deionized water was added, and the solution was stirred for 5 min. Upon complete digestion, the sample was filtered in a 0.45 *μ*m syringe filter. The filtered sample was diluted to 1 : 10.

One milliliter of the diluted sample/standard was drawn out and transferred to a test tube in an ice bath. Six milliliters of cold sulfuric acid/sodium tetraborate mixture (4.77 g sodium tetraborate in 1 L of sulfuric acid) was added to the sample. It was mixed thoroughly and kept cool. Tubes were boiled for 5 min at 100°C and then placed immediately in an ice bath. One-tenth milliliters of 0.0125 M m-hydroxydiphenyl (0.15% in 0.5% sodium hydroxide—stored at 4°C in a container wrapped with aluminum foil) was added. The sample was repeatedly mixed in a vortex until it reached room temperature (25°C). The absorbance of the sample was read at 520 nm against a blank after 20 min.

For the standard, 0-100 *μ*g/mL of galacturonic acid in deionized water was used. For the blank, 0.1 mL of 0.5% sodium hydroxide, instead of m-hydroxydiphenyl, was added. In terms of galacturonic acid, the concentration of the total pectic substance was derived from the standard curve.

#### 2.6.4. Fourier Transform Infrared Spectroscopy (FTIR)

Approximately 12.5 mg of pectin was mixed with 250 mg potassium bromide. The mixture was finely pulverized and put into a pellet-forming die. A force was applied manually to form transparent pellets. For background measurement, a pellet holder containing potassium bromide (without pectin) was inserted into the sample chamber (Thermo Scientific, Nicolet NEXUS 670 FTIR, Madison, Wisconsin). Infrared radiation (400-4000 cm^−1^) was bombarded to the sample at a resolution of 4 cm^−1^ with data spacing every 1.928 cm^−1^ for 64 scans. The resulting spectra were used to identify relevant peaks. A commercial (CP Kelco, Genu®, Atlanta, Georgia) slow set low-methoxy pectin (LMP) was also analyzed for comparison.

#### 2.6.5. Particle Size Distribution

Pectin (1% *w*/*v*) was dissolved in deionized water through continuous stirring for 4 h. The resulting solution was adjusted to pH 6 and then subjected to centrifugation at 10,000 × *g* for 15 min. The decantate was separated, sonicated for 5 min to break any possible aggregates, and then diluted to a factor of 1,000 in deionized water. The particle size was then measured at 25°C using the Zetasizer Nano Series (Malvern Instruments Limited, Westborough, MA). A He–Ne laser was used as the light source while an avalanche photodiode (APD) served as the detector.

### 2.7. Evaluation of Thickening Properties

#### 2.7.1. Effect on Whey Protein Isolate (WPI) Solution

The thickening property of PMP was tested in a protein solution. For the effect of mixing ratio, pectin was dissolved in a WPI solution such that the ratios (a) of the concentration of WPI to that of pectin were 5, 10, and 15. The final pH was adjusted to 5, using 0.1-0.5 M of hydrochloric acid or sodium hydroxide.

For the effect of pH, pectin was dissolved in a WPI solution such that the ratio of the concentration of WPI to that of pectin was 10. The final pH was adjusted to 4, 5, and 6 using 0.1 to 0.5 M of hydrochloric acid or sodium hydroxide.

The resulting mixture was homogenized at 3,000 rpm for 30 s. It was subjected to one cycle of freezing and thawing (FT)—equivalent to 24 h of freezing at -18°C and 24 h of thawing at 10°C. A control, composed of a WPI solution without pectin, was prepared for every pH condition. Moreover, incubation at 10°C for 48 h was also done to simulate the condition without freezing.

The resulting complex was carefully transferred to the rheometer's lower plate, and the surplus was removed using a plastic spatula after lowering the head of the rheometer to the trim position. Viscosity testing at varied shear rates (2-200 s^−1^) was performed at 25°C.

#### 2.7.2. In Vitro Digestion of Pectin-Fortified Orange Juice

The pectin effect on the *in vitro* digestion viscosity of orange juice was tested following the protocol used for dietary fibers [14], with slight modifications. Fifteen grams of Simply® Orange juice, containing 2% PMP/LMP (*w*/*v*), was transferred to a 125 mL Erlenmeyer flask with 4 × 1 cm glass beads. Seven milliliters of simulated gastric fluid (SGF: 0.2% sodium chloride (*w*/*w*) in 0.7% hydrochloric acid (*w*/*v*)) was added. SGF also contains pepsin such that the latter's final concentration was 3.2 mg/mL. The final solution was adjusted to pH 2.0 using 0.1-0.5 M hydrochloric acid. It was then incubated in a shaking water bath at 37°C, at a 175 rpm speed, for 2 h to imitate gastric digestion. The viscosity at physiological shear rates [15] was measured before incubation and after 1 and 2 h.

To the solution used in gastric digestion, 2 mL of 750 mM calcium chloride, 4.6 mL of simulated bile fluid (SBF: containing 8 mg/mL bile salts), and 12 mL of simulated intestinal fluid (SIF: 5 mg/mL pancreatin dissolved in 0.5 M sodium phosphate buffer; pH 7.8) were added. The incubation with shaking was continued for 2 h. The viscosity of the solution was measured in the first hour and at the end of the digestion.

## 3. Results and Discussion

### 3.1. Chemical Composition of “Saba” Banana Peel Waste

The major component of fresh “Saba” banana peel waste was water (86%). Other nutritional components, including ash (1.79%), crude fat (1.11%), and crude fiber (0.74%), were found to be minimal in quantity.

### 3.2. Microwave-Assisted Extraction of Pectin

#### 3.2.1. Screening of Significant Extraction Parameters

A factorial experiment was performed to screen and identify the significant parameters for the microwave-assisted extraction of pectin from “Saba” banana peel waste. The yield of crude pectin (data not shown) ranged from 1-10%. The ANOVA (data not shown) showed that the only significant (*p* < 0.05) individual parameter was SLR. In addition, the 2-way interaction between SLR and temperature and the 3-way interaction between temperature, SLR, and pH were found to be significant (*p* < 0.05). The 3-way interaction has the highest level of effect on pectin's yield, followed by the 2-way interaction and SLR, accordingly.

The identified significant parameters are known to be crucial in the extraction process of pectin. For microwave-assisted extraction, the microwave offers a rapid transfer of energy to the solvent. It results in the dipolar rotation of the water molecules and, subsequently, a rapid increase in the system's temperature. The pressure inside the cell of plant material also increases, destroying the cell wall and allowing the release of the components of the plant material [16]. The heat also inactivates pectin esterase, an enzyme that hydrolyzes the methyl ester bond of pectin and converts the latter into pectate and ethanol [17]. Thus, the pectin concentration for potential extraction by an appropriate solvent, which was diluted hydrochloric acid, was higher.

The diluted hydrochloric acid is a polar solvent and can efficiently absorb microwave energy [18], providing the necessary heat for the extraction of pectin from “Saba” banana peel waste. The corresponding pH of the solvent, whose interaction with other parameters was found significant, provided the system's acidic nature and initiated the hydrolysis of pectin from the protopectin [5]. The optimum pH for pectin extraction ranged from 1-3 [19, 20] in related studies.

The significance of the SLR and its interaction with other parameters was due to the surface area's effect on the extraction. At lower SLR, the surface area is large, and an efficient mass transfer is expected. This is due to the improved contact of the plant material with the extracting solvent [21].

#### 3.2.2. Optimization and Validation of Optimum Microwave-Assisted Extraction Parameters

The three parameters (temperature, SLR, and pH) identified in the factorial experiment were considered for the optimization study. A CCD (Table 1) was used to generate the combination of parameters because it provides a relatively high-quality prediction over the entire design space and does not require the use of points outside the original range [22]. Results showed that the range of microwave-extracted pectin (MEP) was from 0.3 to 14%, with the maximum yield higher than that of the factorial experiment. Upon fitting the results to a second-order equation, a quadratic model was generated. Based on ANOVA (data not shown), the temperature and pH were significant (*p* < 0.05) individual parameters. Their 2-way interaction was also significant (*p* < 0.05) and the square of SLR.

**Figure 1:**
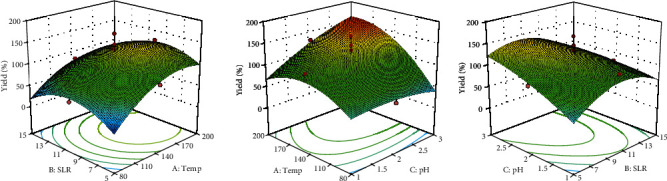
Response surface model for the microwave-assisted extraction of pectin from “Saba” banana peel waste.

The response surface model (Figure 1) shows that the pectin yield increased as the temperature increased up to around 140°C-190°C. Beyond that, the yield started to decrease. This trend was uniform regardless of the temperature's interaction with other parameters. It is possible that at the lower extreme, the temperature is not enough for effective disruption of the plant tissues, while at the upper extreme of the temperature range, possible hydrolysis of pectin and actual degradation of the source material happened. A related study on the effect of microwave on pectin extraction from orange skin using dilute hydrochloric acid reported a destructive effect of the generated heat on the structure of orange skin organization, resulting in the splitting of the cells [16].

The level of effect of pH on the pectin yield was lower than that of temperature. In general, the pectin yield decreased as the acidity of the extracting solvent increased, except when the temperature of the extracting solvent was at the upper extreme.

The model's equation in terms of coded parameters is shown in the following equation

(3) $$\begin{array}{c} \%\text{Yield}=9.62+2.52\text{A}+1.54\text{C}+2.05\text{AC}–2.90{\text{B}}^{2}, \end{array}$$

where A is the temperature, B is the SLR, and C is the pH. The model predicted that the optimal process condition was 8% (*w*/*v*) SLR, pH 3 HCl, and 195°C, with a yield of 12.8%. Upon validation, the yield was 14.20 ± 0.01% or an 11% increase from the predicted yield. The obtained yield was greater than the microwave-extracted pectin from passion fruit (13%) [6] but lower than that of jackfruit rind (17%) [8] and sour orange peel (38%) [23].

Upon characterization, the purity of MEP was found to be 8.83%. Upon repurification (redissolution and reprecipitation) of the MEP, purified microwave-extracted pectin (PMP) was obtained. The PMP has a 25.78% purity. As expected, the increase in the purity resulted in decreased yield—from 14.2% to 4.8%. The said value is lower than that of the previously mentioned passion fruit, jackfruit rind, and sour orange peel.

### 3.3. Characteristics of Pectin

#### 3.3.1. Chemical Composition

Results showed that the equivalent weight of MEP (approx. 2,300 g/eq) was higher than its purified counterpart, PMP (1,500 g/eq). Due to MEP's crude nature, it might contain other groups that contributed to the pectin's unit weight relative to the replaceable hydrogen. Moreover, PMP's lower equivalent weight may be attributed to a higher partial degradation during the extraction [24]. In terms of the methoxyl content, both MEP (0.16%) and PMP (2.01%) were within the normal range (0.1-7%) for low-methoxy pectin [25].

The total pectic content, which determines pectin's purity, was based on the appearance of a chromagen when uronic acid (upon heating at 100°C with sulfuric acid/tetraborate) was reacted with meta-hydroxydiphenyl. Results showed that the purity of MEP and PMP were 9% and 26%, respectively.

The protein contents for MEP and PMP were 0.5% and 1.4%, respectively. The amount of protein in pectin is an important consideration for some food applications, particularly in an emulsion. The protein acts as the anchor between the pectin and the formed oil droplets in an emulsion, resulting in a more stable system [26].

#### 3.3.2. FTIR Profile

**Figure 2:**
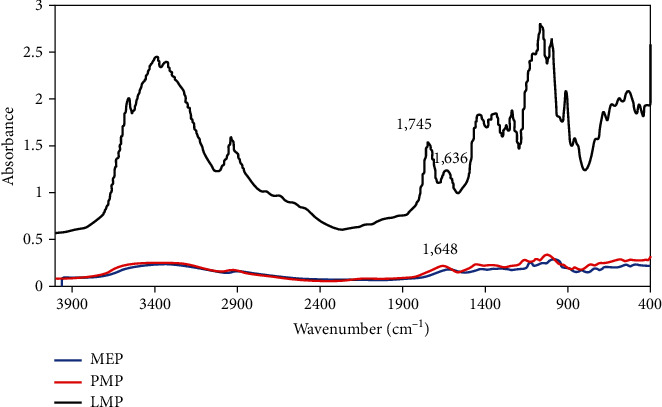
FTIR spectra of different pectin (MEP: microwave-extracted pectin; PMP: purified MEP; LMP: commercial low-methoxy pectin).

For the analysis of the FTIR, bands from 1800-1500 cm^−1^ were considered. This is the pectin's fingerprint region, where relevant functional groups absorb energy from the infrared source [7].  Figure 2 shows that both the MEP and PMP exhibited absorption peaks at around 1630-1600 cm^−1^, which accounts for the carboxyl group's symmetrical stretching vibration. A peak at around 1740 cm^−1^, which describes the C=O bond's stretching in both the ester and carboxyl groups, was also present in LMP but not in MEP and PMP. These findings confirmed the result of the characterization that LMP (7.61%) has more methoxyl groups than MEP (0.16%) and PMP (2.01%). Despite the differences, all three pectins' methoxyl contents fall within the normal range for low-methoxy pectin, which is 0.1-7% [25].

#### 3.3.3. Particle Size Distribution

The particle size of materials, such as food ingredients, is particularly important in the food industry. Knowledge of particle sizes influences the production and handling of ingredients and the formulation, processing, and quality control of food and beverage products. Among the several methods for particle size determination, this study employed dynamic light scattering. The principle is based on a Brownian movement, wherein the fine particles in constant random thermal motion diffuse at a speed related to their size. The smaller the particles are, the faster is the diffusion.

**Figure 3:**
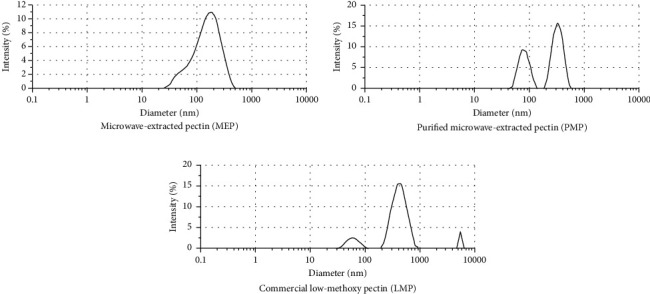
Particle size distribution of 1% (*w*/*v*) pectin extracted from “Saba” banana peel waste.

Figure 3 shows that among the pectins, only the MEP possessed a nonsymmetrical distribution of particle sizes. MEP's size ranged from 25-200 nm, with a mode (the most frequently found size) of around 200 nm. Meanwhile, the PMP exhibited a symmetrical distribution and diversity of sizes ranging from 35-150 nm and 200-600 nm, with a 300 nm mode. LMP, on the other hand, has three ranges of particle size: 30-100 nm, 200-900 nm, and 5 *μ*m-6.5 *μ*m.

### 3.4. Thickening Property of Pectin

#### 3.4.1. Effect of Pectin on the Viscosity of WPI Solution

**Figure 4:**
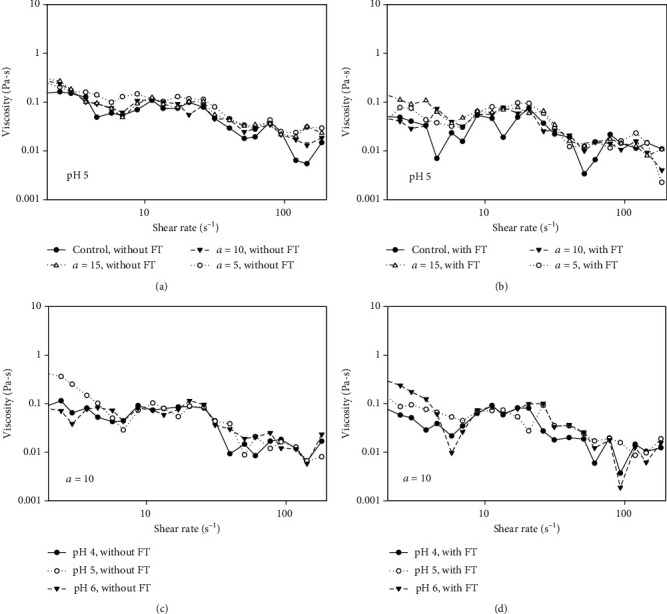
Effect of mixing ratio (a, b) and pH (c, d) on the viscosity of a whey protein isolate—purified microwave-extracted pectin complex with and without freezing and thawing (FT).

Upon measurement of the apparent viscosities, the results (Figure 4) showed that all WPI-PMP mixtures exhibited a shear-thinning property—a decrease in viscosity in response to increased shear rates. This rheological property is similar to that of acid-extracted pectin [27] and other carbohydrates such as guar gum [28] and carboxymethyl cellulose [29].

The mixing ratio and pH of a pectin-protein complex affect the rheological behaviour of a fluid. In this study, varying the shear rates did not generalize the effects of the mixing ratio and pH on the viscosity. However, under specific shear rates, the differences in the viscosities can be evaluated. Hence, a food process with a known shear rate can identify an appropriate mixing ratio and pH to achieve the desired viscosity of a fluid. On the effect of storage conditions, freezing and thawing resulted in almost the same viscosity of the complex as that of without freezing.

#### 3.4.2. Effect of Pectin on the In Vitro Digestion Viscosity of Orange Juice

**Figure 5:**
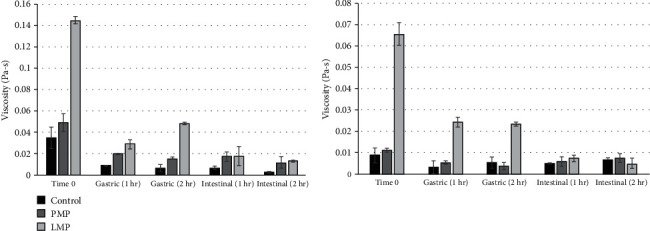
*In vitro* digestion viscosity of pectin (2%)—fortified orange juice at physiologically relevant shear rates (left: ~10 s^−1^, right: ~50s^−1^). Data are mean + standard deviation of three replications.

For the rheology testing of pectin-fortified fruit juice, the viscosity was measured at physiological shear rates:10 and 50 s^−1^ [15], as shown in Figure 5. Before digestion, there was no significant (*p* < 0.05) difference between the apparent viscosity of PMP-fortified orange juice and the control (without PMP). During gastric digestion, both PMP and LMP increased the viscosity of orange juice at a low physiological shear rate. At the same time, only the LMP showed an improved viscosity at a high physiological shear rate. In the remaining hours of the *in vitro* digestion, both pectins improved the juice's viscosity at the low physiological shear rate. Still, they exhibited the same viscosity as the control at a high shear rate. This finding agrees with the results of a related study wherein commercial brands of pectin were found to increase the viscosity of orange juice [30].

A study correlating food viscosity with satiety suggested that fullness during a meal is higher for highly viscous food [31]. Hence, aside from improving the texture of the juice, the addition of PMP could increase the level of fullness during meals. For health-conscious individuals, this could be a potential diet option in maintaining healthy body weight.

## 4. Conclusions

This study showed that microwave irradiation could be used to assist the extraction of pectin from “Saba” banana peel waste. Moreover, the model obtained to predict the optimum microwave-assisted extraction condition was valid, as shown by the close agreement between the predicted (12.8%) and actual yield (14.2%) of pectin. The identified significant parameters (and corresponding optimum conditions) were temperature (195°C), SLR (8% (*w*/*v*)), and pH (3, HCl). Further purification of the microwave-extracted pectin (MEP) resulted in a lower yield (5%) but higher-quality pectin (PMP, 36% purity).

The rheological study showed that the WPI-PMP complex exhibits a shear-thinning property. Moreover, the effects of both mixing ratio and pH can only be evaluated at specific shear rates. On pectin's effect on the *in vitro* digestion viscosity of orange juice, an improvement of viscosity was observed at low shear rate digestion. Overall, the pectin extracted using the optimized microwave-assisted extraction has shown potential for food applications as a thickener.

## Figures and Tables

**Table 1:** Yield based on the central composite design (using coded values) for the microwave-assisted extraction of pectin from “Saba” banana peel waste.

Run	Temperature	Solid-liquid ratio	pH	Yield, % (based on dried peel)
1	-1	-1	-1	1.29 ± 0.01
2	+1	-1	-1	0.28 ± 0.01
3	-1	+1	-1	0.99 ± 0.02
4	+1	+1	-1	3.40 ± 0.02
5	-1	-1	+1	1.13 ± 0.01
6	+1	-1	+1	13.5 ± 0.03
7	-1	+1	+1	0.67 ± 0.01
8	+1	+1	+1	6.07 ± 0.01
9	-1	0	0	3.67 ± 0.01
10	+1	0	0	9.76 ± 0.01
11	0	-1	0	6.55 ± 0.01
12	0	+1	0	6.28 ± 0.02
13	0	0	-1	8.54 ± 0.01
14	0	0	+1	8.53 ± 0.01
15	0	0	0	8.17 ± 0.01
16	0	0	0	10.14 ± 0.01
17	0	0	0	12.53 ± 0.01
18	0	0	0	10.99 ± 0.01
19	0	0	0	7.78 ± 0.04
20	0	0	0	9.38 ± 0.01

## Data Availability

Data other than presented in this paper are available upon request. Please send all communications to Dr. Joel P. Rivadeneira at jprivadeneira@up.edu.ph.

## References

[1] FAO (2020). *Banana market review: February 2020 snapshot*.

[2] PSA (2019). *Supply Utilization Accounts (SUA) of Selected Agricultural Commodities 2016-2018*.

[3] Flórez N., Conde E., Dominguez H. (2015). Microwave assisted water extraction of plant compounds. *Journal of Chemical Technology and Biotechnology*.

[4] Mohapatra D., Mishra S. (2011). *Current trends in drying and dehydration of foods*.

[5] Sandarani M. J. D. C. (2017). A review: different extraction techniques of pectin. *Journal of Pharmacognosy & Natural Products*.

[6] Seixas F. L., Fukuda D. L., Turbiani F. R. B. (2014). Extraction of pectin from passion fruit peel (Passiflora edulis f.flavicarpa) by microwave-induced heating. *Food Hydrocolloids*.

[7] Hosseini S. S., Khodaiyan F., Yarmand M. S. (2016). Optimization of microwave assisted extraction of pectin from sour orange peel and its physicochemical properties. *Carbohydrate Polymers*.

[8] Koh P. C., Leong C. M., Noranizan M. A. (2014). Microwave-assisted extraction of pectin from jackfruit rinds using different power levels. *International Food Research Journal*.

[9] Adetunji L. R., Adekunle A., Orsat V., Raghavan V. (2017). Advances in the pectin production process using novel extraction techniques: a review. *Food Hydrocolloids*.

[10] AOAC (2000). *Official Methods of Analysis*.

[11] Rangana S. (1995). *Handbook of Analysis and Quality Control for Fruit and Vegetable Products*.

[12] Walker J. M. (2002). *The Proteins Protocol Handbook*.

[13] Blumenkrantz N., Asboe-Hansen G. (1973). New method for quantitative determination of uronic acids. *Analytical Biochemistry*.

[14] Fabek H., Messerschmidt S., Brulport V., Goff H. D. (2014). The effect of *in vitro* digestive processes on the viscosity of dietary fibres and their influence on glucose diffusion. *Food Hydrocolloids*.

[15] Borwankar R. P. (1992). Food texture and rheology: a tutorial review. *Journal of Food Engineering*.

[16] Zhongdong L., Guohua W., Yunchang G., Kennedy J. F. (2006). Image study of pectin extraction from orange skin assisted by microwave. *Carbohydrate Polymers*.

[17] Liu Y. (2014). Starch-pectin matrices for encapsulation of ascorbic acid.

[18] Maran J. P., Priya B. (2015). Ultrasound-assisted extraction of pectin from sisal waste. *Carbohydrate Polymers*.

[19] Castillo-Israel K. A. T., Baguio S. F., Diasanta M. D. B., Lizardo R. C. M., Dizon E. I., Mejico M. I. F. (2015). Extraction and characterization of pectin from Saba banana [Musa ‘saba’ (Musa acuminata x Musa balbisiana)] peel wastes: a preliminary study. *International Food Research Journal*.

[20] Krishna Murthy T. P., Bhavya S. G., Mamatha M., Mathew B. B., Dammalli M. (2015). Optimization of microwave assisted extraction of phenolic compounds from *Decalepis hamiltonii* root using response surface methodology. *International Research Journal of Pharmacy*.

[21] Chia V. V., Pang S. F., Gimbun J., Abdullah S., Yusoff M. M. (2019). Effect of amplitude on ultrasonic assisted extraction of caffeic acid from *Andrographis paniculate*. *International Journal of Engineering and Advanced Technology*.

[22] National Institute of Standards and Technology/SEMATECH (2013). *Engineering statistics handbook*.

[23] Hosseini K., Yarmand *Optimization of microwave assisted*.

[24] Yadav S. D., Bankar N. S., Waghmare N. N., Shete D. C. Extraction and characterization of pectin from sweet lime.

[25] Featherstone S. (2015). *Jams, jellies, and related products, in Woodhead Publishing series in food science, technology and nutrition, a complete course in canning and related processes (fourteenth edition)*.

[26] Leroux J., Langendorff V., Schick G., Vaishnav V., Mazoyer J. (2003). Emulsion stabilizing properties of pectin. *Food Hydrocolloids*.

[27] Rivadeneira J. P., Wu T., Gaban P. J. V., Castillo-Israel K. A. T. (2020). Rheological behaviour of purified banana peel pectin from ’ Saba ’ banana [*Musa BBB saba (Musa acuminata x Musa balbisiana)*] peel applied to beverage. *Journal of Advanced Research in Fluid Mechanics and Thermal Sciences*.

[28] Hussain M., Bakalis S., Gouseti O., Zahoor T., Anjum F. M., Shahid M. (2015). Dynamic and shear stress rheological properties of guar galactomannans and its hydrolyzed derivatives. *International Journal of Biological Macromolecules*.

[29] Benchabane A., Bekkour K. (2008). Rheological properties of carboxymethyl cellulose (CMC) solutions. *Colloid and Polymer Science*.

[30] Logan K., Wright A. J., Goff H. D. (2015). Correlating the structure and in vitro digestion viscosities of different pectin fibers to in vivo human satiety. *Food & Function*.

[31] Marciani L., Gowland P. A., Spiller R. C. (2001). Effect of meal viscosity and nutrients on satiety, intragastric dilution, and emptying assessed by MRI. *American Journal of Physiology. Gastrointestinal and Liver Physiology*.

